# ATF6 Alleviates Endothelial Inflammation Following Extended Hepatectomy Through Inhibition of TRIM10/NF‐κB Signaling

**DOI:** 10.1096/fj.202402197RRR

**Published:** 2025-08-13

**Authors:** Cheng‐Cheng Shi, Dong‐Jing Yang, Yang Bai, Binhui Zhou, Yao‐Hui Sun, Xiao‐Ran Xu, Wen‐Zhi Guo, Shui‐Jun Zhang, Yinming Liang, Yang Jin, Ji‐Hua Shi

**Affiliations:** ^1^ Department of Pharmacy The First Affiliated Hospital of Zhengzhou University, Zhengzhou University Zhengzhou China; ^2^ Department of Hepatobiliary and Pancreatic Surgery, Henan Key Laboratory of Digestive Organ Transplantation & Zhengzhou Key Laboratory for HPB Diseases and Organ Transplantation The First Affiliated Hospital of Zhengzhou University Zhengzhou China; ^3^ Laboratory of Genetic Regulators in the Immune System, Henan Collaborative Innovation Center of Molecular Diagnosis and Laboratory Medicine Xinxiang Medical University Xinxiang China; ^4^ The First Clinical School of Medicine of Zhengzhou University Zhengzhou China; ^5^ Department of Biosciences University of Oslo Oslo Norway

**Keywords:** liver sinusoidal endothelial cell, postoperative hepatic failure, tripartite motif‐containing protein 10, unfolded protein response activating transcription factor 6

## Abstract

The inflammatory response in liver sinusoidal endothelial cells (LSECs) is crucial to the pathophysiology of postoperative hepatic failure. The unfolded protein response (UPR) in LSECs following surgical stress exerts an important mechanism for resolving endothelial inflammation and re‐establishing liver homeostasis. We employed 80% hepatectomy in mice to simulate extended hepatectomy and verified the gene expression in the patients who underwent marginal hepatectomy. The UPR in LSECs and endothelial inflammation were induced with tunicamycin or lipopolysaccharides in HUVECs to investigate the expression, effect, and regulation of activating transcription factor 6 (ATF6) in endothelial inflammation. We found that UPR protein ATF6 in LSECs was upregulated and activated following extended hepatectomy in both mice and humans; ATF6 deficiency in mice by either global knockout or LSECs‐specific knockdown failed to alleviate the inflammatory response and led to severe liver injury; genetic knockout or pharmacological inhibition of ATF6 by the ATF6 antagonist Ceapin‐A7 in HUVECs led to severe inflammation through the nuclear factor‐κB (NF‐κB) signaling pathway, while the ATF6 agonist AA147 ameliorated inflammation. Mechanistically, ATF6 induced negative transcriptional control of tripartite motif‐containing protein 10 (TRIM10) and the downstream NF‐κB signaling pathway, thereby suppressing endothelial inflammation. Taken together, our data identify ATF6 as a suppressor of endothelial inflammation following extended hepatectomy and clarify the underlying regulatory mechanism of the ATF6‐TRIM10/NF‐κB signaling pathway. These findings highlight its potential as a therapeutic target for postoperative hepatic failure.

## Graphical abstract

Activating transcription factor 6 (ATF6) in liver endothelial cells is upregulated and activated following the extended hepatectomy in both humans and rodents. ATF6 deficiency or pharmacological inhibition of ATF6 leads to severe endothelial inflammation in mice and HUVECs. Mechanically, ATF6 induces negative transcription control of tripartite motif‐containing protein 10 (TRIM10) and its downstream nuclear factor‐κB (NF‐κB) signaling pathway, thereby suppressing endothelial inflammation. ATF6 has the potential as a therapeutic target for post‐operative hepatic failure. Created in Biorender (http://BioRender.com/5szxuzf).

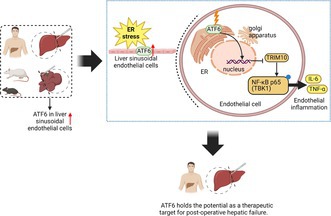

**Abbreviations**
ALT: alanine aminotransferase
AST: aspartate aminotransferase
ATF6: activating transcription factor 6
ChIP: chromatin immunoprecipitation
CHOP: C/EBP homologous protein
ELISA: enzyme‐linked immunosorbent assay
GRP78/HSPA5: glucose‐regulated protein 78
GSEA: Gene Set Enrichment Analysis
HUVECs: human umbilical vein endothelial cells
IL‐6: interleukin‐6
IRE1α: inositol‐requiring transmembrane kinase/endoribonuclease 1α
LPS: lipopolysaccharides
LSECs: liver sinusoidal endothelial cells
NF‐κB: nuclear factor‐κB
PERK: protein kinase R‐like endoplasmic reticulum kinase
PHLF: post‐hepatectomy liver failure
RT‐qPCR: quantitative real‐time polymerase chain reaction
SFSS: small‐for‐size syndrome
STING: stimulator of interferon genes
TBK1: TANK‐binding kinase1
TNF‐α: tumor necrosis factor‐α
TRIM10: tripartite motif‐containing protein 10
TUNEL: Terminal Deoxynucleotidyl Transferase mediated dUTP Nick‐End Labeling
UPR: unfolded protein response

## 1 Introduction

Post‐hepatectomy liver failure (PHLF) and small‐for‐size syndrome (SFSS), characterized by excessive inflammation, uncontrolled injury, and jeopardized regeneration, can occur after extended hepatectomy and reduced‐size liver transplantation, leading to increased postoperative morbidity and mortality rates of up to 40%–60% [1, 2]. To accelerate postoperative parenchymal restoration and improve prognosis, many efforts aiming at decreasing portal vein flow and portal pressure, including portal vein embolization/ligation, have been made to attenuate sinusoidal injury and maintain liver function in the future liver remnant [2]. However, the effectiveness of these clinical interventions is still unclear due to the unresolved mechanisms underlying PHLF and SFSS. The surgical stress response following partial hepatectomy and reduced‐size liver transplantation can activate both hepatocytes and hepatic nonparenchymal cells and promote liver regeneration through the release of cytokines, chemokines, and growth factors [3]. As a hallmark of postoperative liver regeneration, the activation of nonparenchymal cells, including liver sinusoidal endothelial cells (LSECs), Kupffer cells, and hepatic stellate cells, plays a critical role in regulating inflammation and hepatocyte proliferation [3, 4]; in particular, the endothelial inflammation is crucial to the initiation of liver homeostasis and regeneration.

The intense or prolonged endoplasmic reticulum (ER) stress and oxidative stress in hepatic nonparenchymal cells can lead to the unfolded protein response (UPR) and inflammatory responses, both of which are crucial to liver homeostasis and regeneration [5, 6]. In mammalian cells, the UPR is regulated by three ER transmembrane proteins, inositol‐requiring transmembrane kinase/endoribonuclease 1α (IRE1α), protein kinase R‐like ER kinase (PERK), and activating transcription factor 6 (ATF6), in response to ER stress [7, 8]. The IRE1 and PERK arms of the UPR have been reported to induce nuclear factor‐κB (NF‐κB) activation and increase cytokine production after hepatectomy [5]. Our recent study revealed that ATF6 activation during liver ischemia–reperfusion injury induces the protective connective tissue growth factor expression and reduces endothelial inflammation [9]. However, the mechanism by which ATF6 influences postoperative inflammation has not been fully elucidated [8].

Our previous transcriptomic analysis revealed the upregulation of intrahepatic ATF6 expression and activity following extended hepatectomy in rats [10]. Thus, to elucidate the regulatory mechanism of the inflammatory response and liver regeneration involved in PHLF and SFSS, we investigated the expression, function, and regulation of ATF6 following extended hepatectomy in the present study.

## 2 Materials and Methods

### 2.1 Clinical Study of Patients After Partial Hepatectomy

The human study protocol was approved by the Research Committee of the Affiliated Hospital of Zhengzhou University (registration number: 2019‐KY‐21) and conformed to the ethical guidelines of the 1975 Declaration of Helsinki [11]. A total of 14 patients who underwent open partial hepatectomy for liver cancer and provided informed consent were retrospectively included in the study [10]. According to the resection size, the patients were grouped into two groups: those who underwent marginal hepatectomy with a resection size greater than 45% (Group M, *N* = 8) and those who underwent minor hepatectomy (wedge and segment resection) with a resection size less than 30% (Group C, *N* = 6). The distant non‐tumor liver tissue was obtained at 1‐h postsurgery to verify the selected targets from preclinical analysis in rodents.

### 2.2 Animals and Development of Partial Hepatectomy in Rodents

The animal experimental protocol was approved and supervised by the Ethics Committee of The First Affiliated Hospital of Zhengzhou University (registration number: 2019‐KY‐183) and was performed in accordance with the ARRIVE guidelines. Twelve‐week‐old male Sprague–Dawley rats and C57BL/6J mice (Keli Experimental Animal Center, Beijing, China) were bred and maintained in compliance with the protocols outlined in our previous studies [9, 12]. Genetic ablation of ATF6 in mice was achieved by knocking out the ATF6 gene (ATF6‐KO) in fertilized C57BL/6J eggs via the CRISPR/Cas9 system [9, 13]. Polymerase chain reaction (PCR) was used to validate the protein expression of the selected mutants (Figure S1).

Based on our previous study involving 30% to 80% hepatectomy in rodents [10, 14], we performed hepatectomy with a resection size of 80% (PH80) to simulate extended hepatectomy in rats and mice, and hepatectomy with a resection size of 70% (PH70) was used as the standard regeneration model. The animals in the present study were divided into two cohorts. In the detection cohort, a total of 33 rats were grouped into the PH70 (70% major hepatectomy, *N* = 15), PH80 (80% extended hepatectomy, *N* = 15) and sham (sham control, *N* = 3) groups. Blood and tissue samples were harvested from 3 rats in each group at 0 h (control), 1, 6, 12, 24, and 72 h after partial hepatectomy. Total RNA from the liver was isolated with TRIzol reagent (Invitrogen, Carlsbad, CA, USA) for further high‐throughput sequencing.

In the validation cohort, liver tissues, which were obtained at specific time points following 70% major hepatectomy in wild‐type (WT) mice and 80% extended hepatectomy in WT mice (*N* = 5 at each time point) and ATF6 knockout mice (ATF6‐KO, N = 5 at each time point), were used to assess parameters of hepatic injury, apoptosis, liver regeneration, and to determine the expression and function of the differentially expressed genes by western blotting, immunohistochemistry, immunofluorescence, and Terminal Deoxynucleotidyl Transferase mediated dUTP Nick‐End Labeling (TUNEL) assay. Blood samples were obtained from the vena cava to measure the levels of alanine aminotransferase (ALT), aspartate aminotransferase (AST), tumor necrosis factor‐α (TNF‐α) and interleukin‐6 (IL‐6) by enzyme‐linked immunosorbent assay (ELISA). ATF6‐KO mice were generated using the Clustered Regularly Interspaced Short Palindromic Repeats (CRISPR)—CRISPR associated protein 9 (Cas9) system [9]. For LSECs‐specific ATF6 knockdown, adeno‐associated viruses (AAV)‐based delivery system with pAAV‐TIE1p‐MCS‐miR30shRNA (Atf6)‐WPRE or pAAV‐TIE1p‐MCS‐miR30shRNA (NC)‐WPRE (0.5 × 10^12^ vg, OBiO Technology, Shanghai, China) were injected by tail vein injection to C57BL/6 mice. Three weeks after viral injection, gene detection and 80% extended hepatectomy were employed (*N* = 5 per group at each time point). Knockout and knockdown of ATF6 in mice were verified by polymerase chain reaction (PCR) and western blotting (Figures S1 and S2). In addition, hematoxylin and eosin staining and histological analysis were employed to evaluate viral toxicity caused by viral injection (Figure S2).

### 2.3 Cell Lines and Gene Editing

Human umbilical vein endothelial cells (HUVECs) were purchased from Shanghai Cell Bank (Shanghai Cell Bank, Chinese Academy of Sciences, Shanghai, China). HUVECs were cultured according to the procedures recommended by the Shanghai Cell Bank and maintained at 37°C and 5% CO_2_ in a humidified incubator. ER stress in HUVECs was induced with tunicamycin (10 mg/mL, B7417, Apexbio, Shanghai, China), and endothelial inflammation was stimulated with lipopolysaccharides (LPS, L7770, 10 μg/mL, Sigma‐Aldrich, Luxembourg) and/or 10 μM ATF6 antagonist Ceapin‐A7/ATF6 agonist AA147.

A jetPRIME transfection system (PolyPlus, Shanghai, China) and plasmids (pLVX‐mNeonGreen‐nTAF6, pCMV‐dR8.2 dvpr and pCMV‐VSV‐G) were used to generate ATF6‐overexpressing (ATF6‐OE) HUVECs. ATF6 knockout (ATF6‐KO) HUVECs were generated using the CRISPR‐Cas9 system [9, 15]. siRNAs against ATF6 and tripartite motif‐containing protein 10 (TRIM10) were synthesized by GenePharma (Shanghai, China) and transfected with jetPRIME transfection reagent (PolyPlus, Shanghai, China) (Table S2) [16]. ATF6 expression in HUVECs was detected and validated by PCR and western blotting.

### 2.4 Transcriptome Sequencing

Total mRNA was extracted from the rat liver samples or HUVECs according to the instructions for the mirVana mRNA Isolation Reagent (Ambion, USA). Transcriptome sequencing and bioinformatics analysis were performed in collaboration with OE Biotech Co. Ltd. (Shanghai, China) using the Illumina HiSeq X Ten platform (Illumina Inc., San Diego, CA, USA). The project and the raw transcriptome sequencing data have been registered and uploaded to the National Center for Biotechnology Information (PRJNA687124).

### 2.5 Immunohistochemistry, Immunofluorescence and TUNEL Assay

Immunostaining, including immunohistochemistry and immunofluorescence, was performed on formalin‐fixed liver tissue specimens as described previously [12]. Immunostaining was conducted with primary antibodies against ATF6 (1:100, ab122897, Abcam, Cambridge, UK), Ki‐67 (1:200, ab15580, Abcam, Cambridge, UK), LSEC marker CD31 (1:100, 11265‐1‐AP, Proteintech, Wuhan, China), macrophage marker CD68 (1:100, 25747‐1‐AP, Proteintech, Wuhan, China), T helper cell marker CD4 (1:100, 65104‐1‐AP, Proteintech, Wuhan, China), and TRIM10 (1:200, bs‐9409R, Bioss, Beijing, China). The immunohistochemistry index was derived by multiplying the intensity score by the area score. TUNEL label staining was performed on paraffin‐embedded liver sections using a Cell Apoptosis Detection Kit (G1502, Servicebio, Wuhan, China). TUNEL‐positive nuclei were counted per high‐power field by immunofluorescence.

### 2.6 Quantitative Real‐Time PCR (RT–qPCR)

RNA extraction, cDNA synthesis, and quantitative PCR were performed as described previously [10, 12]. The primers for the target genes were designed and synthesized by Shanghai Generay Biotech Co. Ltd. (Table S2). Quantitative analysis of gene expression was performed on an ABI Prism 7000 (Applied Biosystems, Foster City, CA, USA) using 2 × SYBR Green master mix (Applied Biosystems, Foster City, CA, USA). Gene expression is presented relative to the level of the housekeeping gene β‐actin.

### 2.7 Western Blot

Protein extracts of liver tissue were prepared and processed for western blot analysis as previously described [10, 12]. The primary antibodies used were anti‐ATF4 (1:1000, 10835‐1‐AP, Proteintech, Wuhan, China), X‐box binding protein 1 (Xbp1, 1:1000, 24868‐1‐AP, Proteintech, Wuhan, China), glucose‐regulated protein 78 (GRP78, 1:1000, WL03157, Wanleibio, Shenyang, China), GRP94 (1:1000, 14700‐1‐AP, Proteintech, Wuhan, China), ATF6 (1:1000, 24169‐1‐AP, Proteintech, Wuhan, China), TRIM 10 (1:1000, bs‐9409R, Bioss, Beijing, China), nuclear factor‐κB (NF‐κB) p65 (1:1000, bs‐20160R, Bioss, Beijing, China), phospho‐NF‐κB p65 (1:1000, ser536, bs‐0982R, Bioss, Beijing, China), and glyceraldehyde‐3‐phosphate dehydrogenase (GAPDH, 1:5000, 60004‐1‐Ig, Proteintech, Wuhan, China). Immunoreactivity was visualized using secondary horseradish peroxidase‐conjugated rabbit (1:5000, SA00001‐2, Proteintech, Wuhan, China) or mouse (1:5000, SA00001‐1, Proteintech, Wuhan, China) antibodies, and ECL Western Blotting Substrate (P10100, NCM Biotech, Suzhou, China). Quantification of the target protein was performed by dividing the target protein density by GAPDH density. For phospho‐NF‐κB, quantification was performed relative to total NF‐κB protein.

### 2.8 Chromatin Immunoprecipitation (ChIP) Assay

The ChIP assay was performed according to the protocol [9, 12] provided by the ChIP Assay Kit (KT101‐01, GZSCBIO, Guangzhou, China). After stimulation with LPS, the HUVEC lysates were immunoprecipitated with a ChIP‐grade antibody against ATF6 (sc‐166659, Santa Cruz, Dallas, USA) or an anti‐rabbit IgG (KT101‐01, GZSCBIO, Guangzhou, China) as the negative control. The interaction of ATF6 with the predicted promoter of TRIM10 was detected by RT–qPCR. The detailed sequences of primers used for TRIM10 are shown in Table S2.

### 2.9 Luciferase Assay

A dual‐luciferase reporter assay was performed as previously described [9, 12] to confirm the direct interaction between the ATF6α:NF‐YA heterodimer and the promoter of TRIM10. The detailed sequences of primers used for TRIM10 are shown in Table S2. The psicheck2‐TRIM10 vector, a psicheck2 construct containing the Renilla luciferase reporter, and a psicheck2 control were transfected into HUVECs. After stimulation with LPS, luciferase activity was measured with the Dual Luciferase Reporter Assay System (E1910, Promega, Beijing, China). The firefly luciferase activity was normalized to the Renilla luciferase activity.

### 2.10 Enzyme‐Linked Immunosorbent Assay (ELISA)

The serum TNF‐α, IL‐6, ALT, and AST levels were measured by ELISA according to the manufacturers' protocols (C009‐2‐1, C010‐2‐1, Nanjing Jiancheng Bioengineering Institute, Nanjing, China; EK282/4‐96, EK206/3‐96, Multiscience Biotech, Hangzhou, China).

### 2.11 Statistical Analysis

The treatment effects were compared by two‐tailed Student's *t* test for comparisons between two groups and one‐way analysis of variance for comparisons between multiple groups. The statistical analysis was performed with SPSS 21.0 (IBM, Armonk, NY, USA). A probability level of less than 5% (*p* < 0.05) was considered to indicate statistical significance.

## 3 Results

### 3.1 Intrahepatic ATF6‐Mediated UPR Is Activated Following Extended Hepatectomy

**FIGURE 1:**
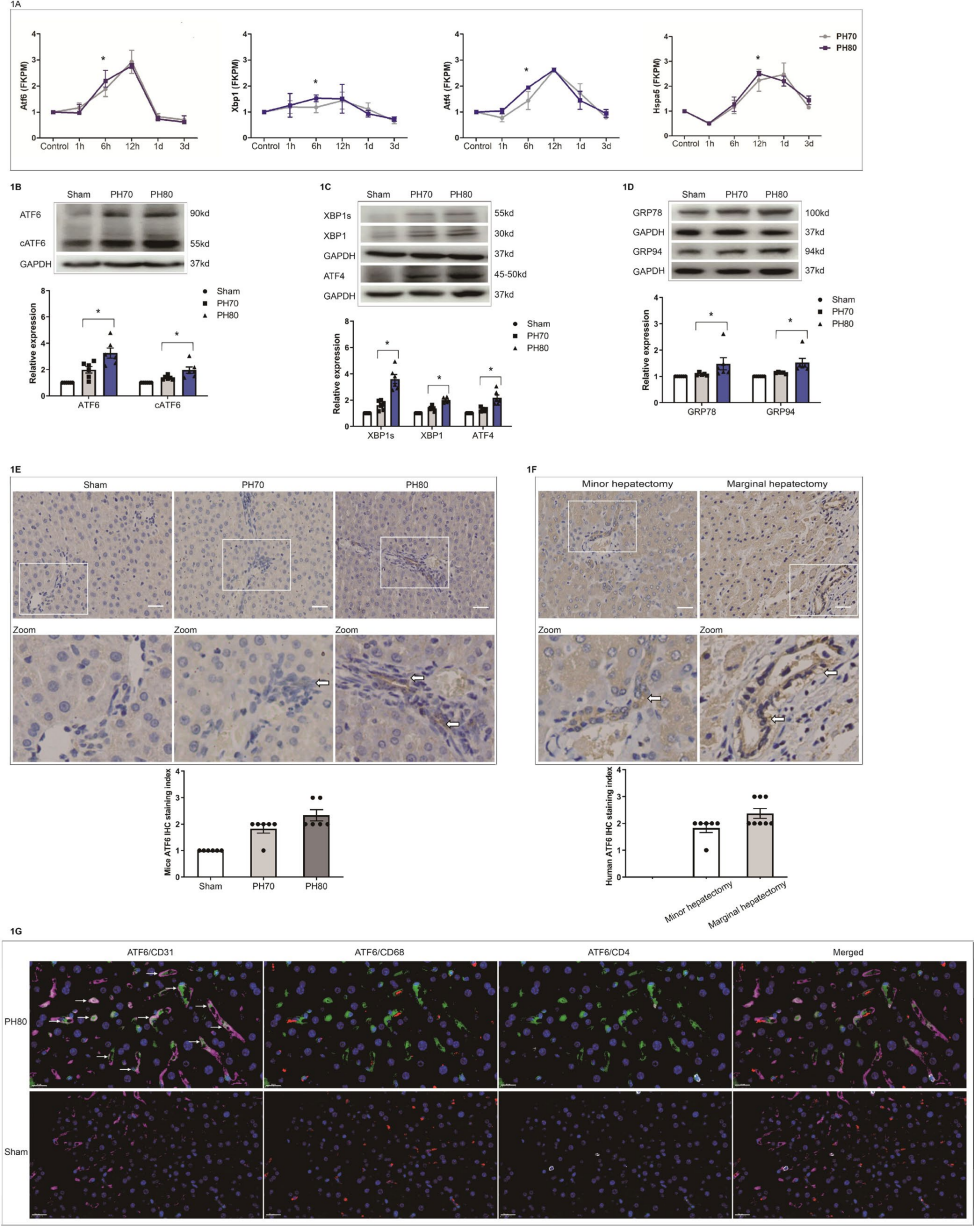
Expression of unfolded protein response (UPR) genes following extended hepatectomy (PH80) and major hepatectomy (PH70). (A) Transcriptomic analysis of the expression of UPR genes (atf6, atf4, xbp1, and hspa5/GRP78) at 0 h (control), 1 h, 6 h, 12 h, 1 day, and 3 days after PH80 and PH70 in rats (*p < 0.05 between PH80 and PH70, *N* = 3 at each time point); (B) the expression of ATF6 and cleaved ATF6 (cATF6) following PH80 and PH70 in mice determined by western blot analysis and semiquantitative analysis (*N* = 5). (C) Western blot analysis of the expression of XBP1, spliced XBP1 (XBP1s) and ATF4 following PH80 and PH70 in mice and semiquantitative analysis (*N* = 5); (D) western blot analysis of GRP78 and GRP94 expression following PH80 and PH70 treatment in mice and semiquantitative analysis of GRP78 and GRP94 expression (*N* = 5); (E) immunohistochemical analysis of hepatic ATF6 expression following PH80 and PH70 treatment in mice (original magnification ×400, scale bars: 50 μm) and semiquantitative analysis of the results (*N* = 5); (F) assessment of hepatic ATF6 expression following marginal hepatectomy (*N* = 8) and minor hepatectomy (*N* = 6) in humans by immunohistochemistry (original magnification ×400, scale bars: 50 μm) and semiquantitative analysis; (G) hepatic ATF6 expression (green) in liver sinusoidal endothelial cells (CD31, purple), macrophages (CD68, orange) and Th cells (CD4, white) in the early stage after extended hepatectomy in mice by immunofluorescence (original magnification ×400, scale bars: 20 μm).

To explore the involvement of the UPR in the process after extended hepatectomy, we reanalyzed the transcriptomic data from our previous study on small‐for‐size livers following extended hepatectomy in rats [10]. Transcriptomic analysis revealed that the mRNA expressions of UPR proteins, including Atf4, Xbp1, Atf6, and heat shock protein family A member 5 (Hspa5) were significantly upregulated at 6–12 h following extended hepatectomy compared with major hepatectomy (*p* < 0.05, Figure 1A). Consistent with these findings, Western blot analysis confirmed that the induction of ATF4, the spliced form of XBP1s, cleaved ATF6 (cATF6), ATF6‐induced GRP78 (HSPA5), and GRP94 in liver tissues from extended hepatectomy compared to major hepatectomy (*p* < 0.05, Figure 1B–D).

To investigate the cell‐specific expression and activation of ATF6 following extended hepatectomy, we used immunohistochemistry to assess the morphological expression of ATF6 in liver samples from both mice and humans (Figure 1E,F). Notably, immunohistochemistry and semiquantitative analysis revealed that ATF6 expression was significantly and dominantly upregulated in LSECs at 6–12 h following extended hepatectomy (*p* < 0.05, Figure 1E,F). This finding was further corroborated by immunofluorescence staining, which revealed that ATF6 expression and activation following major hepatectomy and extended hepatectomy were dominantly detected in CD31‐positive LSECs, instead of the increased CD68‐positive macrophages or CD4‐positive Th cells (Figures 1G and S3). These above results suggest that ATF6 is dominantly activated in LSECs and may at least partly contribute to the postoperative response following extended hepatectomy.

### 3.2 LSECs‐Specific ATF6 Attenuates the Early Inflammatory Response and Hepatic Injury Following Extended Hepatectomy

The acute inflammatory response plays a pivotal role in postoperative liver physiology. To evaluate the impact of ATF6 on postoperative liver inflammation and injury, we compared the serum levels of proinflammatory cytokines and liver enzymes from ATF6‐KO mice and LSEC‐specific ATF6 knockdown mice with the control mice.

**FIGURE 2:**
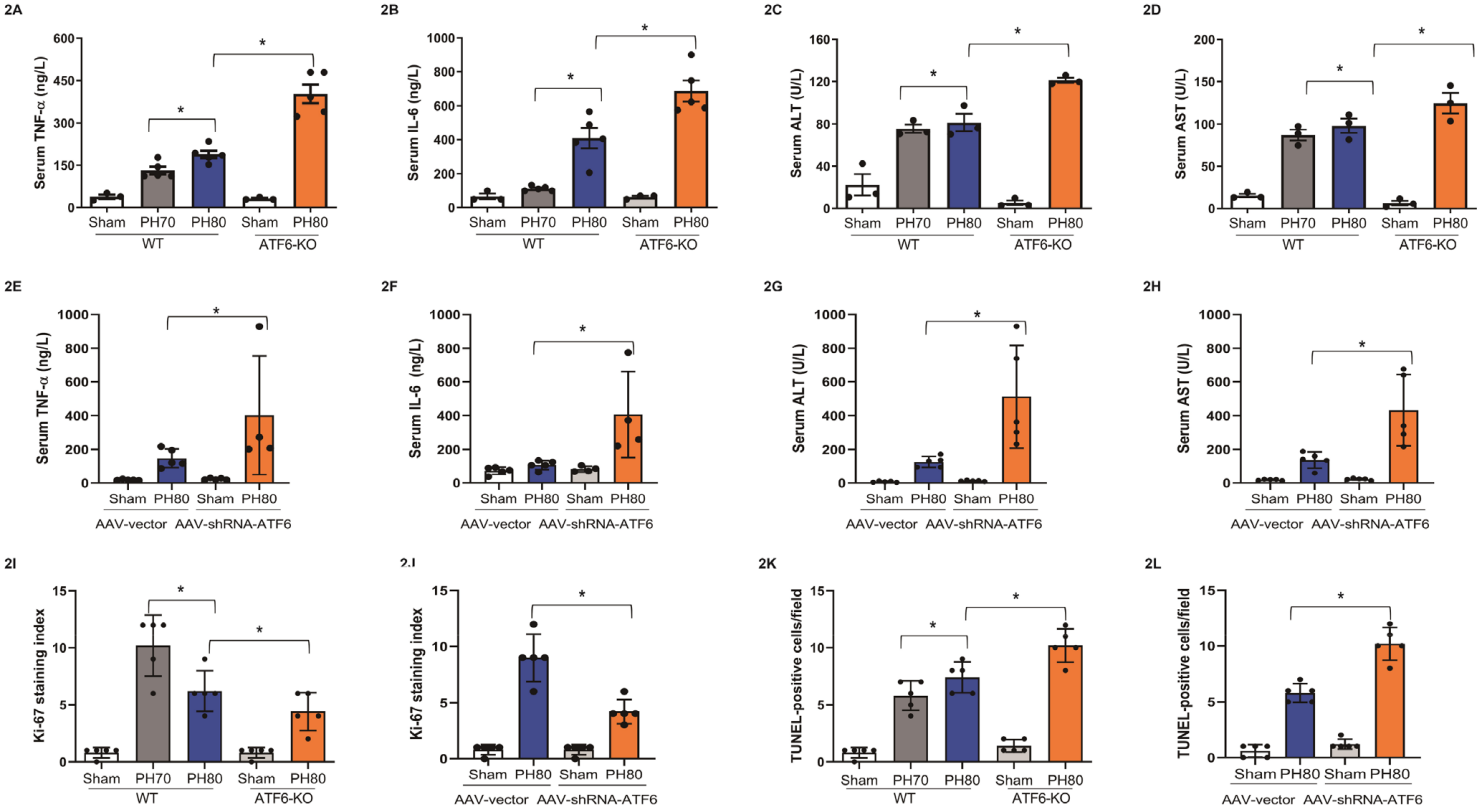
Expression of inflammatory cytokines in the liver and injury parameters following mouse extended hepatectomy (PH80) and major hepatectomy (PH70). (A, B) Serum IL‐6 and TNF‐α levels at 12 h following mouse hepatectomy by ELISA (*p < 0.05, between PH80 and PH70 or between WT and ATF6‐KO mice, *N* = 5); (C, D) serum ALT and AST levels at 12 h following mouse hepatectomy (*p < 0.05, between PH80 and PH70 or between WT and ATF6‐KO mice, *N* = 5); (E, F) serum IL‐6 and TNF‐α levels at 12 h following mouse extended hepatectomy by ELISA (*p < 0.05, between NC and ATF6‐KD mice, *N* = 5); (G, H) serum ALT and AST levels at 12 h following mouse extended hepatectomy (*p < 0.05, between NC and ATF6‐KD mice, *N* = 5); (I) Ki‐67 staining on day 3 after hepatectomy (*p < 0.05, WT vs. ATF6‐KO mice, *N* = 5). (J) Ki‐67 staining on day 3 after hepatectomy (*p < 0.05, NC vs. ATF6‐KD mice, *N* = 5). (K) TUNEL label staining on day 3 after hepatectomy (*p < 0.05, WT vs. ATF6‐KO, *N* = 5). (L) TUNEL staining on day 3 after hepatectomy (*p < 0.05, NC vs. ATF6‐KD mice, *N* = 5).

In the WT mice, the serum TNF‐α and IL‐6 levels were notably elevated at 12 h following extended hepatectomy according to ELISA (*p* < 0.05, Figure 2A,B), and the serum ALT and AST levels increased at 12 h following extended hepatectomy (*p* < 0.05, Figure 2C,D). Compared with the WT mice, the global ATF6 knockout (ATF6‐KO) mice exhibited significantly higher levels of TNF‐α and IL‐6 in the serum, as determined by ELISA (*p* < 0.05, Figure 2A,B), at 6–12 h following extended hepatectomy. Consistent with these findings, the increase in the serum ALT and AST levels was higher at 12 h following extended hepatectomy in the ATF6‐KO mice (*p* < 0.05, Figure 2C,D). In addition, mice with LSECs‐specific ATF6 knockdown with AAV‐based delivery system developed more severe liver inflammation, marked by higher serum levels of TNF‐α and IL‐6, and ALT and AST at 12 h following extended hepatectomy (*p* < 0.05, Figure 2E–H).

Since the acute inflammatory response could influence liver regeneration and apoptosis following extended hepatectomy [4], liver regeneration and hepatic apoptosis on day 3 after hepatectomy were assessed and compared by Ki67 staining and TUNEL assay staining, when both Ki‐67‐positive cells and the TUNEL‐positive cells are pronounced [17, 18]. ATF6 deficiency either by ATF6‐KO mice or LSECs‐specific ATF6 knockdown mice decreased the Ki‐67‐positive cell count (*p* < 0.05, Figures 2I,J and S4) and increased the TUNEL‐positive cell count (*p* < 0.05, Figures 2K,L and S4). Collectively, these findings suggested that activation of LSECs‐specific ATF6 plays a protective role by repressing the early inflammatory response after extended hepatectomy.

### 3.3 ATF6 Suppresses NF‐κB‐Mediated Endothelial Inflammation

**FIGURE 3:**
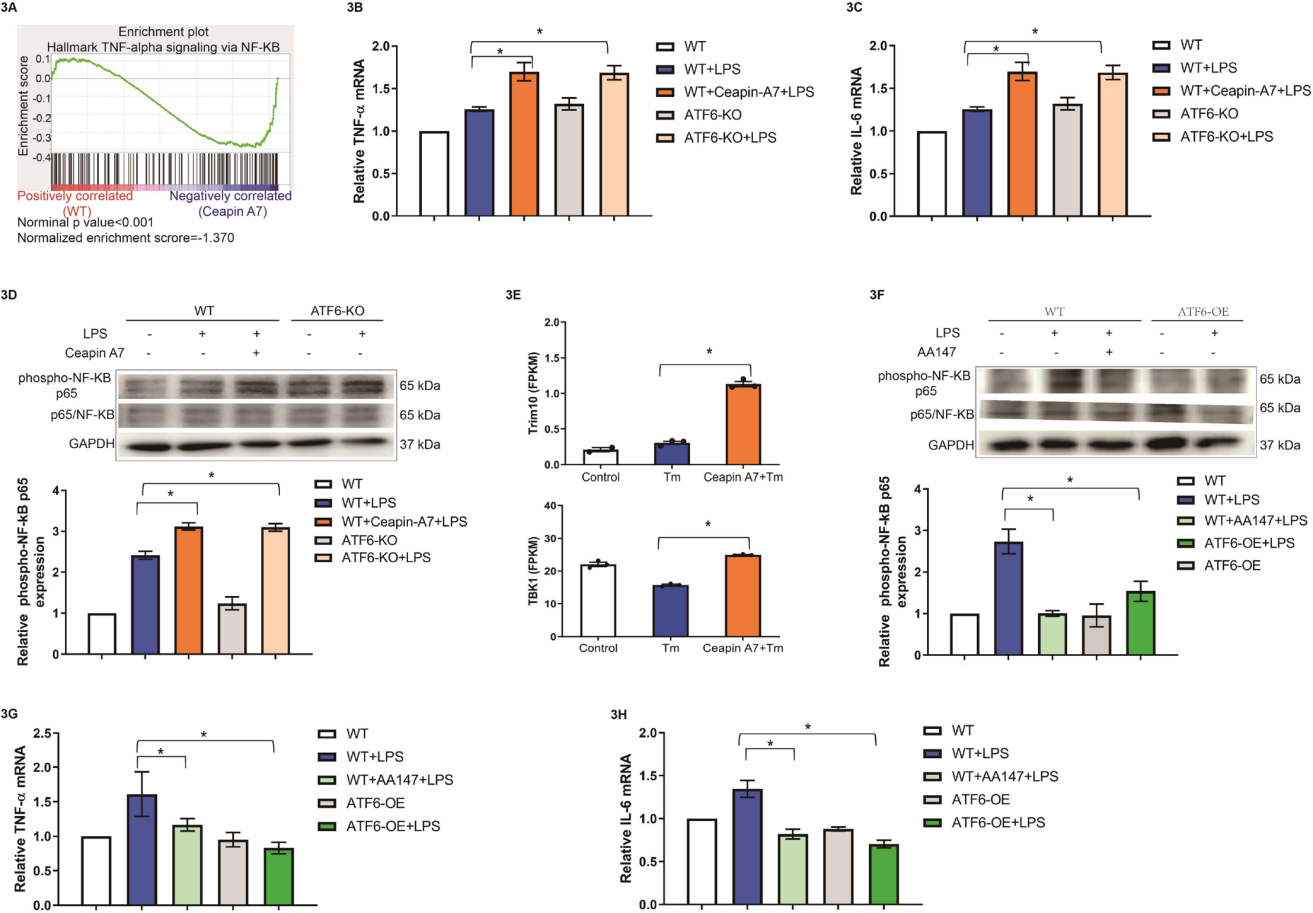
ATF6 suppresses inflammation through the NF‐κB signaling pathway in HUVECs. (A) Gene Set Enrichment Analysis of ATF6 inhibition (10 μM Ceapin‐A7) under 10 mg/mL tunicamycin‐induced UPR in HUVECs revealed that the NF‐κB signaling pathway was activated during ATF6‐mediated inflammation. (B, C) Gene expression of TNF‐α and IL‐6 in WT and ATF6‐knockout HUVECs treated with or without 10 μM Ceapin‐A7 and stimulated with 10 μg/mL LPS determined by RT–qPCR (*p < 0.05, *N* = 3). (D) Western blot analysis of the expression and semiquantitative analysis of phospho‐NF‐κB p65 and NF‐κB p65 in WT and ATF6‐knockout HUVECs treated with or without 10 μM Ceapin‐A7 after stimulation with 10 μg/mL LPS (*p < 0.05, *N* = 4). (E) The expression of tripartite motif‐containing protein 10 (TRIM10) and TANK‐binding kinase 1 (TBK1) after ATF6 inhibition with 10 μM Ceapin‐A7 and stimulation with 10 mg/mL tunicamycin was determined by RNA‐seq analysis (*p < 0.05, *N* = 3). (F) Western blot analysis of the expression and semiquantitative analysis of phospho‐NF‐κB p65 and NF‐κB p65 in WT and ATF6‐overexpressing HUVECs treated with or without 10 μM Ceapin‐A7/AA147 after stimulation with 10 μg/mL LPS (*p < 0.05, *N* = 3). (G, H) Gene expression of TNF‐α and IL‐6 in WT and ATF6‐overexpressing HUVECs treated with or without 10 μM AA147 and stimulated with 10 μg/mL LPS determined by ELISA (*p < 0.05, *N* = 3).

Endothelial inflammation is recognized as a pivotal factor in the initiation and progression of surgery‐induced liver injury and subsequent liver regeneration [19]. To elucidate the involvement of LSECs‐specific ATF6 in the regulation of endothelial inflammation, we conducted a transcriptomic analysis using cultured HUVECs treated with the ER stress inducer tunicamycin in the presence or absence of the ATF6 inhibitor Ceapin‐A7. By gene set enrichment analysis (GSEA), we observed that Ceapin‐A7 activated the NF‐κB signaling pathway in HUVECs (Figure 3A). Accordingly, the upregulated expressions of the downstream target genes, TNF‐α and IL‐6, were confirmed by RT–qPCR after treatment with the ATF6 inhibitor Ceapin‐A7 and after ATF6 KO (*p* < 0.05, Figure 3B,C). In addition, in HUVECs, phospho‐NF‐κB p65 was highly activated in HUVECs treated with ATF6 inhibitor Ceapin‐A7 and in ATF6‐KO HUVECs according to western blot analysis (*p* < 0.05, Figure 3D). Notably, TRIM10, a key activator of the canonical NF‐κB signaling pathway [20, 21], was significantly upregulated (*p* < 0.05, Figure 3E), and the expression of TANK‐binding kinase 1 (TBK1) increased (*p* < 0.05, Figure 3E) after ATF6 activation was inhibited by Ceapin‐A7. Instead, ATF6 agonist AA147 and ATF6 overexpression led to the suppression of phospho‐NF‐κB p65, as shown by western blot analysis (*p* < 0.05, Figure 3F). In addition, the expression levels of TNF‐α and IL‐6 were downregulated after ATF6 agonist AA147 or ATF6 overexpression as determined by RT–qPCR (*p* < 0.05, Figure 3G,H). These above results indicate that ATF6 in LSECs suppresses endothelial inflammation through the negative regulation of the NF‐κB signaling pathway.

### 3.4 ATF6 Regulates the NF‐κB Signaling Pathway Through TRIM10

**FIGURE 4:**
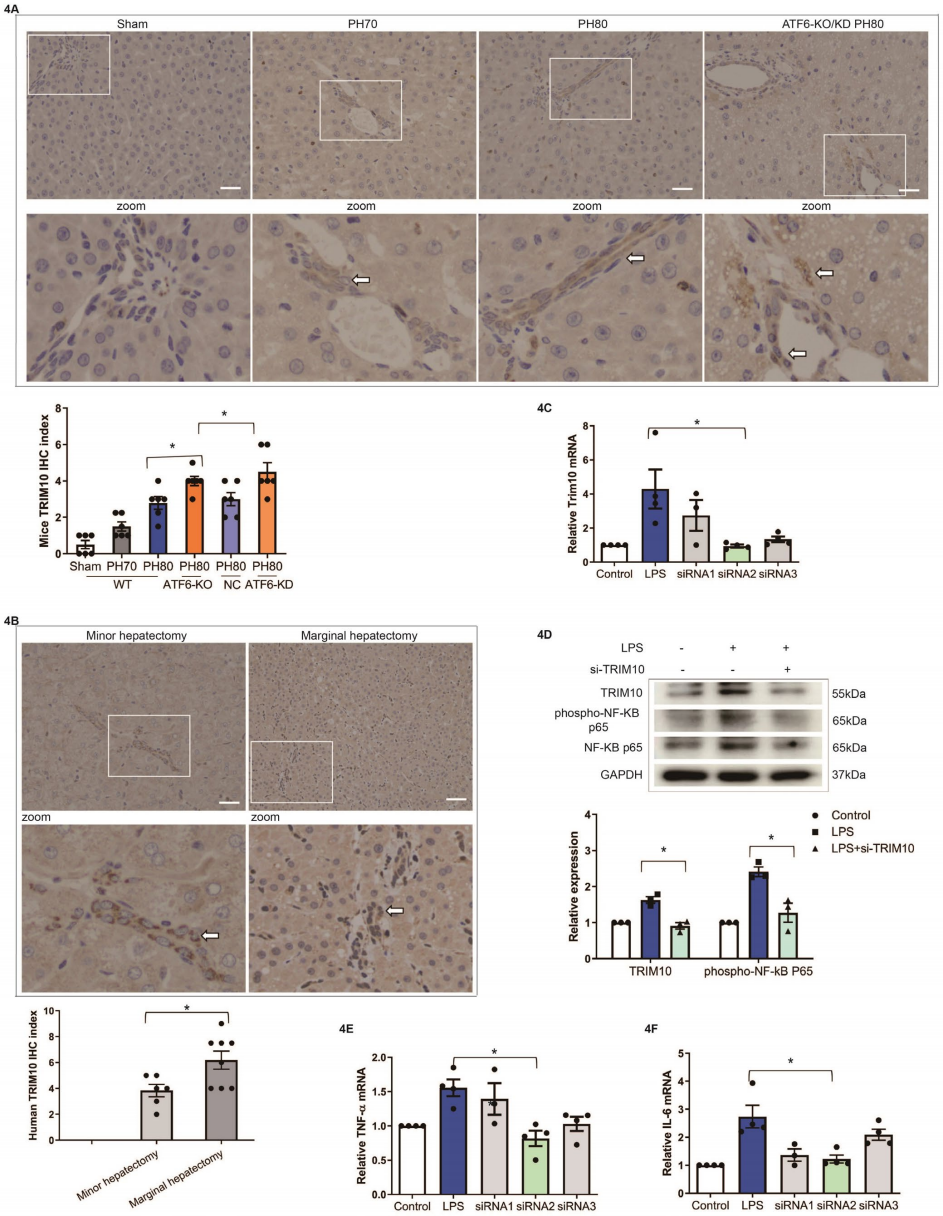
TRIM10 expression following extended hepatectomy (PH80) and major hepatectomy (PH70) and its role in endothelial inflammation. (A) Hepatic TRIM10 expression following PH80 (*N* = 5) and PH70 (*N* = 5) in WT mice, PH80 (*N* = 5) in ATF6‐knockout mice, as well as PH80 (*N* = 5) in LSEC ATF6‐specific deficiency mice, as determined by immunohistochemistry (original magnification ×400, scale bars: 50 μm) and semiquantitative analysis (*p < 0.05); (B) analysis of hepatic TRIM10 expression following marginal hepatectomy (*N* = 8) and minor hepatectomy (*N* = 6) in humans by immunohistochemistry (original magnification ×400, scale bars: 50 μm) and semiquantitative analysis (*p < 0.05). (C) The expression and quantification of TRIM10 in HUVECs with and without TRIM10 siRNA knockdown after stimulation with 10 μg/mL LPS were determined by RT–qPCR (*p < 0.05, *N* = 4). (D) Western blot analysis of the expression and semiquantitative analysis of TRIM10 and phospho‐NF‐κB p65 in HUVECs with and without TRIM10 siRNA knockdown after stimulation with 10 μg/mL LPS (*p < 0.05, *N* = 3). (E, F) Gene expression of TNF‐α and IL‐6 in cells with and without TRIM10 siRNA knockdown after stimulation with 10 μg/mL LPS, as determined by RT–qPCR (*p < 0.05, *N* = 3).

As illustrated above, ATF6 might mediate endothelial inflammation through the TRIM10/NF‐κB signaling pathway. A recent study revealed that TRIM10 ubiquitinates TBK1 and activates the downstream NF‐κB p65 signaling pathway [21]. To investigate the molecular mechanism of ATF6‐mediated endothelial inflammation, we focused on the verification of the effect of TRIM10 on the NF‐κB signaling pathway and the regulatory mechanism of ATF6 on TRIM10. First, the expression of TRIM10 was investigated in both mouse and human samples by immunohistochemistry (Figure 4A,B) and was found to be upregulated in nonparenchymal cells, especially in LSECs, following extended hepatectomy by semiquantitative analysis (*p* < 0.05). In addition, TRIM10 expression in liver tissues was greater in the global ATF6 and LSEC ATF6‐specific deficiency mice following extended hepatectomy (*p* < 0.05, Figure 4A). In cultured human endothelial cells, knockdown of TRIM10 by siRNA reduced phospho‐NF‐κB p65, as determined by RT–qPCR and western blot (*p* < 0.05, Figure 4C,D), and inhibited TNF–α and IL–6, as shown by RT–qPCR (*p* < 0.05, Figure 4E,F), indicating that the TRIM10/NF‐κB signaling pathway mediates endothelial inflammation, and ATF6 regulates the NF‐κB signaling pathway through TRIM10.

### 3.5 ATF6 Suppresses Transcriptional Regulation of TRIM10

**FIGURE 5:**
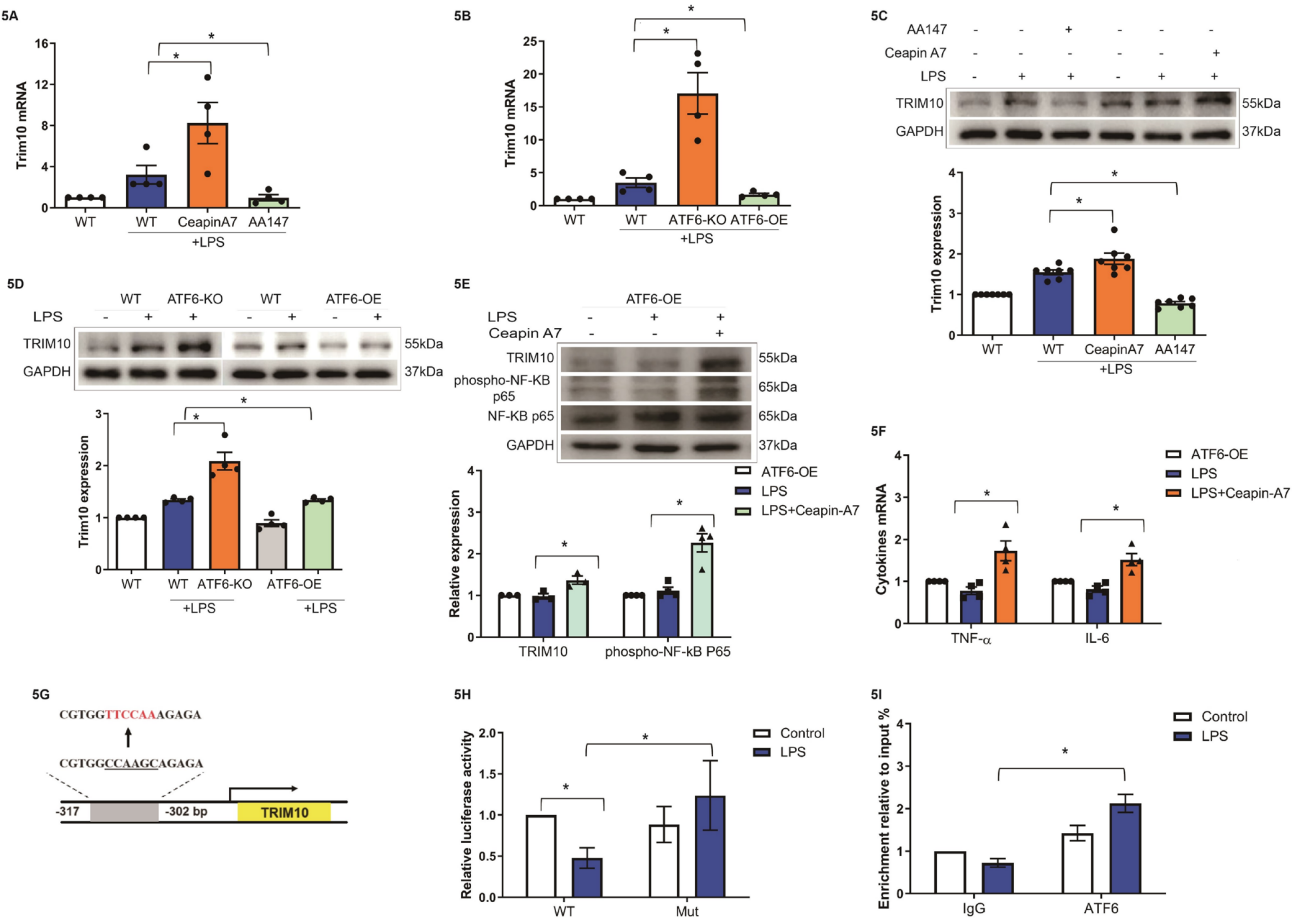
ATF6 regulates TRIM10 transcription in HUVECs. (A) The expression of TRIM10 after stimulation with 10 μM Ceapin‐A7 or AA147 and 10 μg/mL LPS was determined by RT‐qPCR (*p < 0.05, *N* = 3). (B) The expression of TRIM10 after ATF6 knockout or ATF6 overexpression and stimulation with 10 μg/mL LPS was determined by RT‐qPCR (*p < 0.05, *N* = 3). (C) Expression and semiquantitative analysis of TRIM10 after 10 μM Ceapin‐A7 or AA147 treatment and stimulation with 10 μg/mL LPS by western blot (*p < 0.05, *N* = 3); (D) western blot analysis of the expression and semiquantitative analysis of TRIM10 after ATF6 knockout/overexpression in cells stimulated with 10 μg/mL LPS (*p < 0.05, *N* = 3). (E) Western blot analysis of the expression and semiquantitative analysis of TRIM10 and phospho‐NF‐κB p65 in ATF6‐overexpressing HUVECs after 10 μM Ceapin‐A7 stimulation with 10 μg/mL LPS (*p < 0.05, *N* = 3); (F) gene expression of TNF‐α and IL‐6 in ATF6‐overexpressing HUVECs after 10 μM Ceapin‐A7 stimulation with 10 μg/mL LPS, as determined by RT–qPCR (*p < 0.05, *N* = 3). (G) The putative ATF6α:NF‐YA binding sites in the TRIM10 promoter and the nucleotide sequences representing the predicted binding sequences are shown, with the red letters indicating core elements. (H) Luciferase activity of transfected HUVECs transfected with TRIM10 and its mutant construct after treatment with 10 μg/mL LPS determined by dual‐luciferase reporter assay (*p < 0.05, *N* = 4). (I) The binding of ATF6α to the TRIM10 promoter in HUVECs after treatment with 10 μg/mL LPS, as determined by ChIP‐qPCR (*p < 0.05, *N* = 3).

Furthermore, ATF6 knockout or Ceapin‐A7 treatment increased the LPS‐stimulated TRIM10 expression, as determined by RT–qPCR (*p* < 0.05, Figure 5A,B) and western blotting (*p* < 0.05, Figure 5C,D); while ATF6 overexpression or AA147 treatment decreased TRIM10 expression, as determined by RT–qPCR (*p* < 0.05, Figure 5A,B) and western blotting (*p* < 0.05, Figure 5C,D). The reduction in TRIM10, phospho‐NF‐κB p65, and the synthesis of TNF‐α and IL‐6 caused by ATF6 overexpression in HUVECs was rescued by the ATF6 antagonist Ceapin‐A7 (*p* < 0.05, Figure 5E,F).

To determine whether ATF6 represses TRIM10 gene transcription, JASPAR was used to search for putative ATF6α: NF‐YA heterodimer binding sites in the promoter region of the TRIM10 gene. The analysis revealed one ATF6α: NF‐YA binding site (CGTGGCCAAGCAGAGA) in the −317/−302 bp region relative to the transcription start site of the human TRIM10 gene (Figure 5G). A luciferase reporter assay confirmed that the DNA fragment containing this site was responsive to LPS (*p* < 0.05); whereas disruption of the site abrogated the transcriptional response (Figure 5H). To further confirm the binding of ATF6 to this site, a chromatin immunoprecipitation (ChIP) assay was performed and showed that ATF6 enrichment was significantly increased when HUVECs were treated with AA147 (*p* < 0.05, Figure 5I). The results above indicated that ATF6 could suppress the activation of the TRIM10/NF‐κB signaling pathway as a transcriptional suppressor.

## 4 Discussion

During acute liver injury following liver surgery, LSECs—located at the blood‐parenchyma interface—modulate their physicochemical properties and coordinate communication between stress responses and the hepatic microenvironment. This process critically influences the initiation and progression of acute liver injury [3, 22, 23]. Activation of UPR pathways in endothelial cells exacerbates oxidative stress and inflammation, which are pivotal to liver homeostasis, stress responses, and regeneration [6]. Specifically, the IRE1, PERK, and ATF6 UPR branches and their crosstalk can trigger proinflammatory signals post‐hepatectomy [5, 24, 25]. However, the role and regulation of ATF6 in postoperative inflammation remain incompletely defined [8].

Our previous study using rodent 80% hepatectomy (simulating extended resection) [10] demonstrated ATF6 upregulation and activation predominantly in LSECs. Both global and LSEC‐specific ATF6 deficiency exacerbated post‐resection outcomes: heightened liver inflammation, impaired regeneration, and increased apoptosis. Conversely, ATF6 overexpression or agonist treatment in HUVECs suppressed inflammatory injury, while ATF6 deficiency in HUVECs led to increased inflammatory injury. Transcriptome sequencing and GSEA revealed that ATF6 knockout or pharmacological inhibition in HUVECs activated NF‐κB signaling by negatively regulating TRIM10, a key activator of NF‐κB p65. These findings indicate that LSEC‐expressed ATF6 acts as an anti‐inflammatory suppressor postoperatively, suggesting its therapeutic potential for PHLF and SFSS.

Interestingly, several studies have reported that ATF6 primed and exacerbated the pro‐inflammatory liver injury through the C/EBP homologous protein (CHOP) and Toll‐like receptor pathways [24, 25]. Thus, the ATF6‐mediated UPR may play dual regulatory roles in liver inflammation and homeostasis. On the one hand, as a protective mechanism, ATF6 can restore ER homeostasis and protect cells from protein aggregates. On the other hand, overwhelming stimuli exceed the regulatory capacity of the ER, and downstream ATF6‐mediated UPR signaling can aggravate liver inflammatory injury and induce cell death [5, 6]. The underlying mechanism may be that the broad effects of ATF6 differ depending on the liver cell type and phase of liver inflammation [6, 11]. In addition, the transcription factor ATF6 has diverse functions in liver inflammation and liver injury and may function through different downstream targets. The mechanism of the ATF6‐mediated UPR following surgical stress is still worthy of further investigation.

Our earlier study demonstrated that ATF6 is beneficial for liver functional recovery against warm and cold ischemia injury in a rat normothermic mechanical perfusion model and a mouse liver ischemia–reperfusion injury model [9, 26]. However, overall ATF6 activation and its effect on endothelial barrier function and inflammation have still not been fully elucidated [27]. This study elucidates ATF's protective mechanism against endothelial inflammation via the TRIM10/NF‐κB axis. TRIM10 (a ubiquitin E3 ligase) catalyzes ubiquitination of stimulator of interferon genes (STING), facilitating STING‐TBK1 complex formation and subsequent NF‐κB activation [21]. We confirmed that TRIM10 depletion attenuates NF‐κB signaling and inflammation, validating its role as a positive regulator of TBK1/NF‐κB [21].

One limitation of this study is that the current findings were obtained in a preclinical model in healthy rodents. In clinical settings, most patients are diagnosed with underlying chronic liver disease. Liver metabolic dysfunction, fibrosis, and cancer progression have been shown to correlate with the activation of UPR pathways including ATF6 [28, 29, 30]. The effect and mechanism of the ATF6‐mediated UPR following surgical stress on the course of liver fatty diseases and hepatocarcinogenesis are worthy of further investigation. Another major concern is that the ATF6 expression in liver macrophages and the other immune cells is rare but may be vital for the development and resolution of postoperative inflammation. The current study revealed the expression and function of ATF6 in LSECs. ATF6‐mediated UPR in the hepatic immune cells needs further research.

## 5 Conclusion

The major UPR sensor ATF6 is upregulated and activated in LSECs following extended hepatectomy and functions at least partly as a suppressor of postoperative inflammation; the mechanism underlying this suppressive effect is related to the negative transcriptional control of the TRIM10/NF‐κB signaling pathway. Thus, these findings highlight ATF6 as a therapeutic factor for PHLF and SFSS.

## Author Contributions

Conceptualization, Ji‐Hua Shi and Yang Jin; investigation, methodology, data curation, and formal analysis, Cheng‐Cheng Shi, Dong‐Jing Yang, Yang Bai, Binhui Zhou, Yao‐Hui Sun, and Xiao‐Ran Xu; funding acquisition, Ji‐Hua Shi and Yang Jin; project administration, Wenzhi Guo, Shuijun Zhang, and Ji‐Hua Shi; resources, Binhui Zhou, Wen‐Zhi Guo, Shui‐Jun Zhang, Yinming Liang, and Ji‐Hua Shi; supervision, Wenzhi Guo, Shui‐Jun Zhang, Yang Jin, and Ji‐Hua Shi; validation and visualization: Cheng‐Cheng Shi, Dong‐Jing Yang, Yang Bai, and Xiao‐Ran Xu; writing – original draft, Cheng‐Cheng Shi, Dong‐Jing Yang, and Yang Bai; writing – review and editing, Wen‐Zhi Guo, Shui‐Jun Zhang, Yinming Liang, Yang Jin, and Ji‐Hua Shi.

## Conflicts of Interest

The authors declare no conflicts of interest.

## Supporting information


**Figure S1:** Strategy and PCR detection of ATF6‐KO (knockout) mice.

**Figure S2:** Detection of ATF6 expression by western blot (*p < 0.05, *N* = 5) and histological analysis by hematoxylin and eosin staining (original magnification ×100, scale bars: 50 μm) in the LSECs' ATF6‐specific KD (knockdown) mice.

**Figure S3:** Intrahepatic expressions of LSECs (CD31, yellow), macrophage (CD68, red) and Th (CD4, green) following extended hepatectomy in WT (A) and ATF6‐KO (B) mice by immunofluorescence (original magnification ×630, scale bars: 20 μm, arrow indicating the specific cells with positive staining).

**Figure S4:** Liver regeneration was assessed by Ki67 IHC staining (A and B), and hepatic apoptosis was detected by TUNEL label staining (C and D) after hepatectomy (original magnification ×400, scale bars: 20 μm).

**Table S1:** fsb270933‐sup‐0005‐Tables.docx.

## Data Availability

The data are available on request from the corresponding author.
