# Supplementary material for: ATF6 Alleviates Endothelial Inflammation Following Extended Hepatectomy Through Inhibition of TRIM10/NF‐κB Signaling
Source: FASEB J. 2025 Aug 13;39(16):e70933. doi: 10.1096/fj.202402197RRR (PMC12344622; doi:10.1096/fj.202402197RRR)

Figure S1

Strategy for detecting gene knockout mice

Primer

oGE723 GTGTGTGCATGTAGAGACCAGAG
oGE724 TACTCCTTAGGTTGCACTGGTCA
oGE725 CACTGGGGAAGCAGAGAAAGATG

Oligo	WT	KO targeted
oGE723+oGE724	no amplicon	~585 bp
oGE723+oGE725	367bp	no amplicon

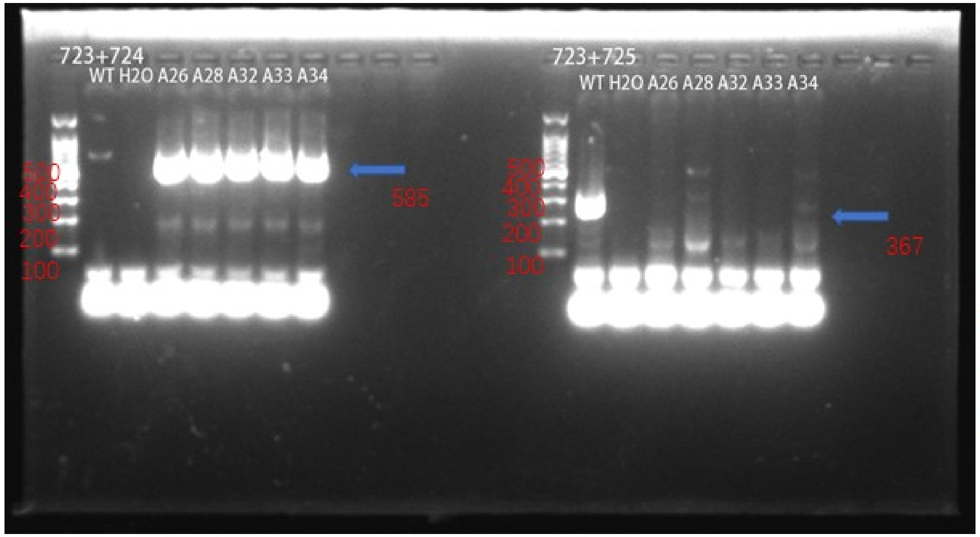

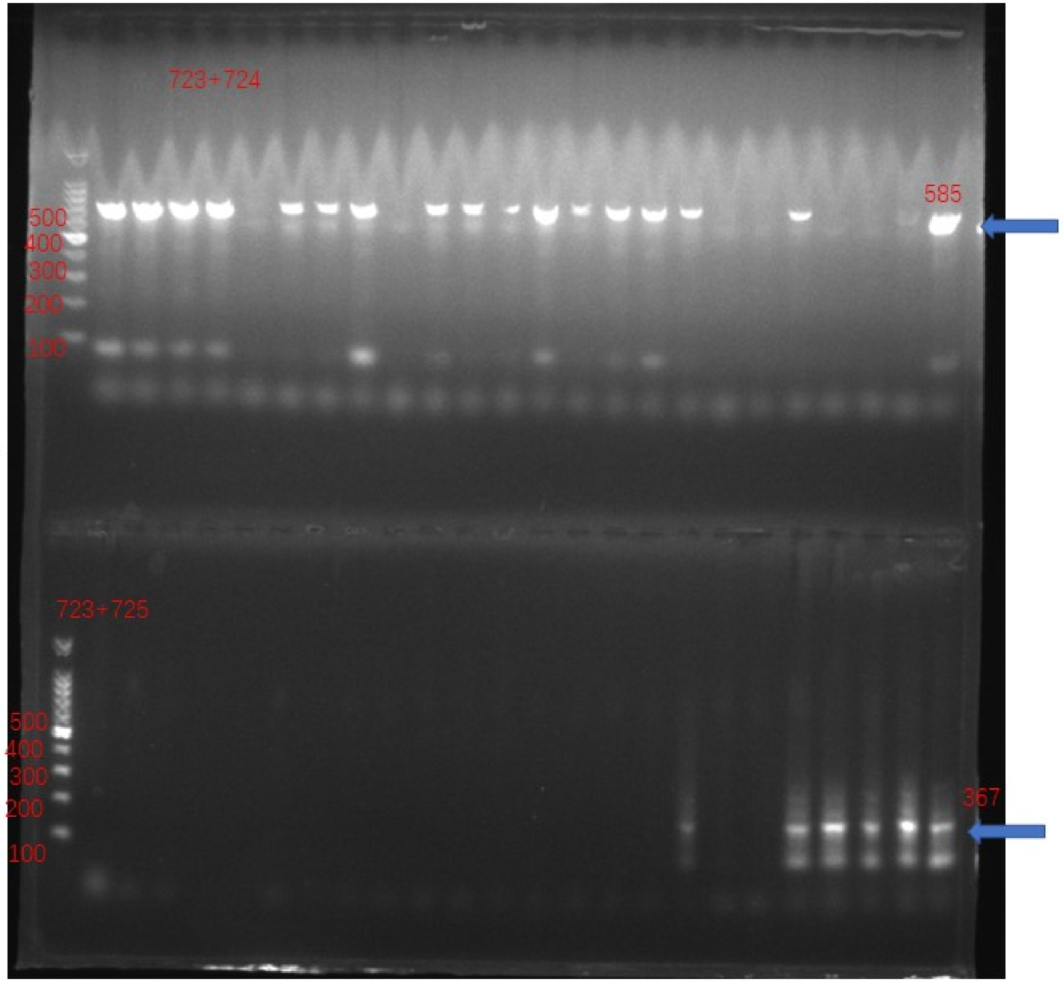

Supplement: Supplementary file 1 — Figure S1: Strategy and PCR detection of ATF6‐KO (knockout) mice. [file FSB2-39-e70933-s005.pdf]
