# Supplementary figures and images for: ATF6 Alleviates Endothelial Inflammation Following Extended Hepatectomy Through Inhibition of TRIM10/NF‐κB Signaling
Source: FASEB J. 2025 Aug 13;39(16):e70933. doi: 10.1096/fj.202402197RRR (PMC12344622; doi:10.1096/fj.202402197RRR)

Figure S2

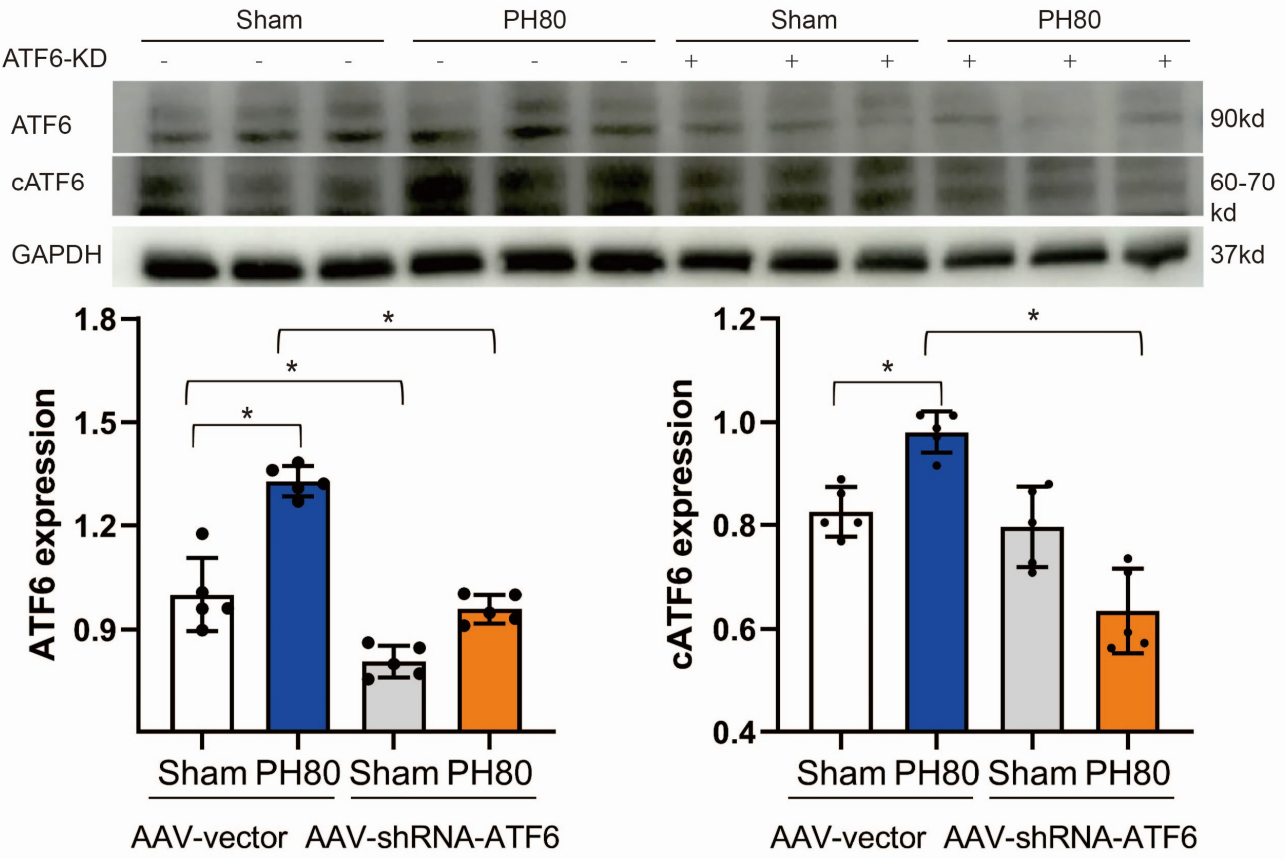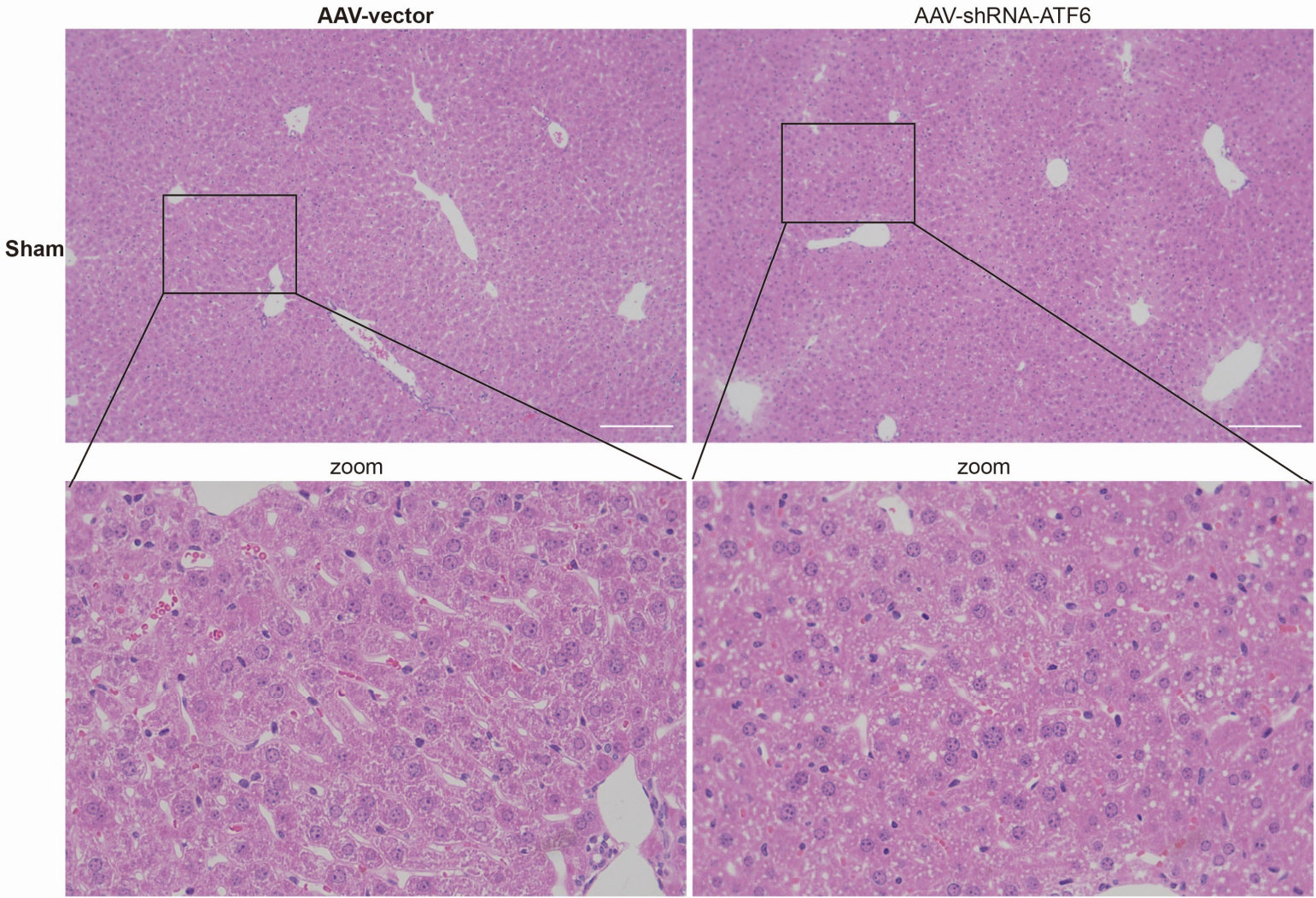

Supplement: Supplementary file 2 — Figure S2: Detection of ATF6 expression by western blot (*p < 0.05, N = 5) and histological analysis by hematoxylin and eosin staining (original magnification ×100, scale bars: 50 μm) in the LSECs' ATF6‐specific KD (knockdown) mice. [file FSB2-39-e70933-s003.pdf]

Figure S3

A

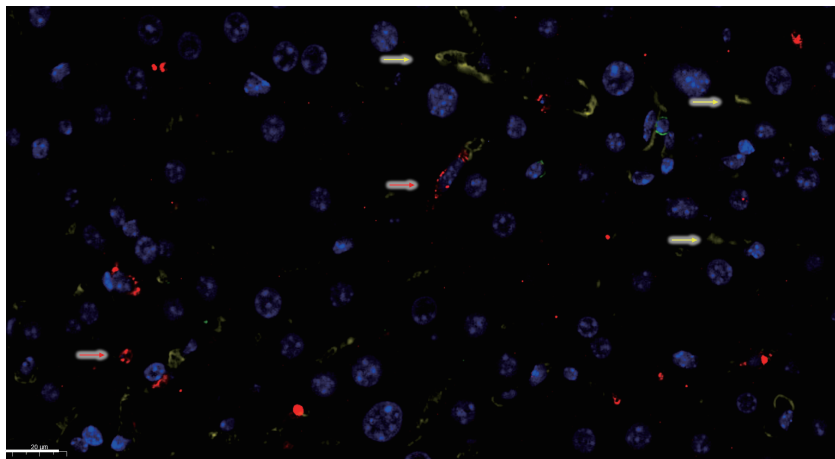

B

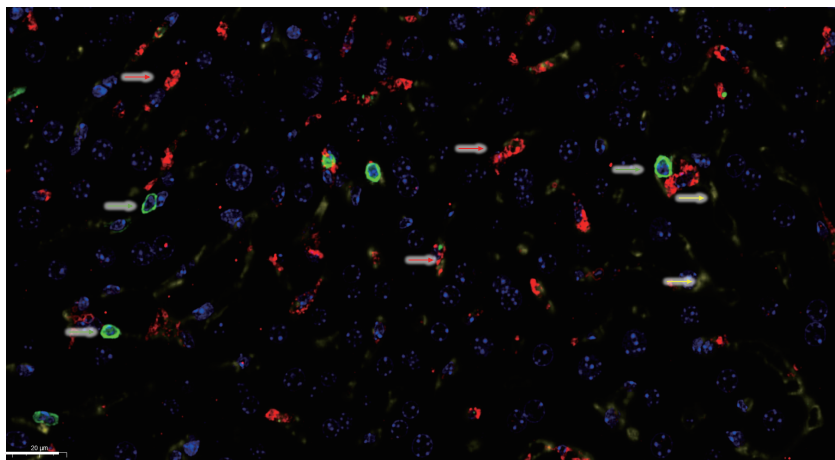

Supplement: Supplementary file 3 — Figure S3: Intrahepatic expressions of LSECs (CD31, yellow), macrophage (CD68, red) and Th (CD4, green) following extended hepatectomy in WT (A) and ATF6‐KO (B) mice by immunofluorescence (original magnification ×630, scale bars: 20 μm, arrow indicating the specific cells with positive staining). [file FSB2-39-e70933-s001.pdf]

Figure S4

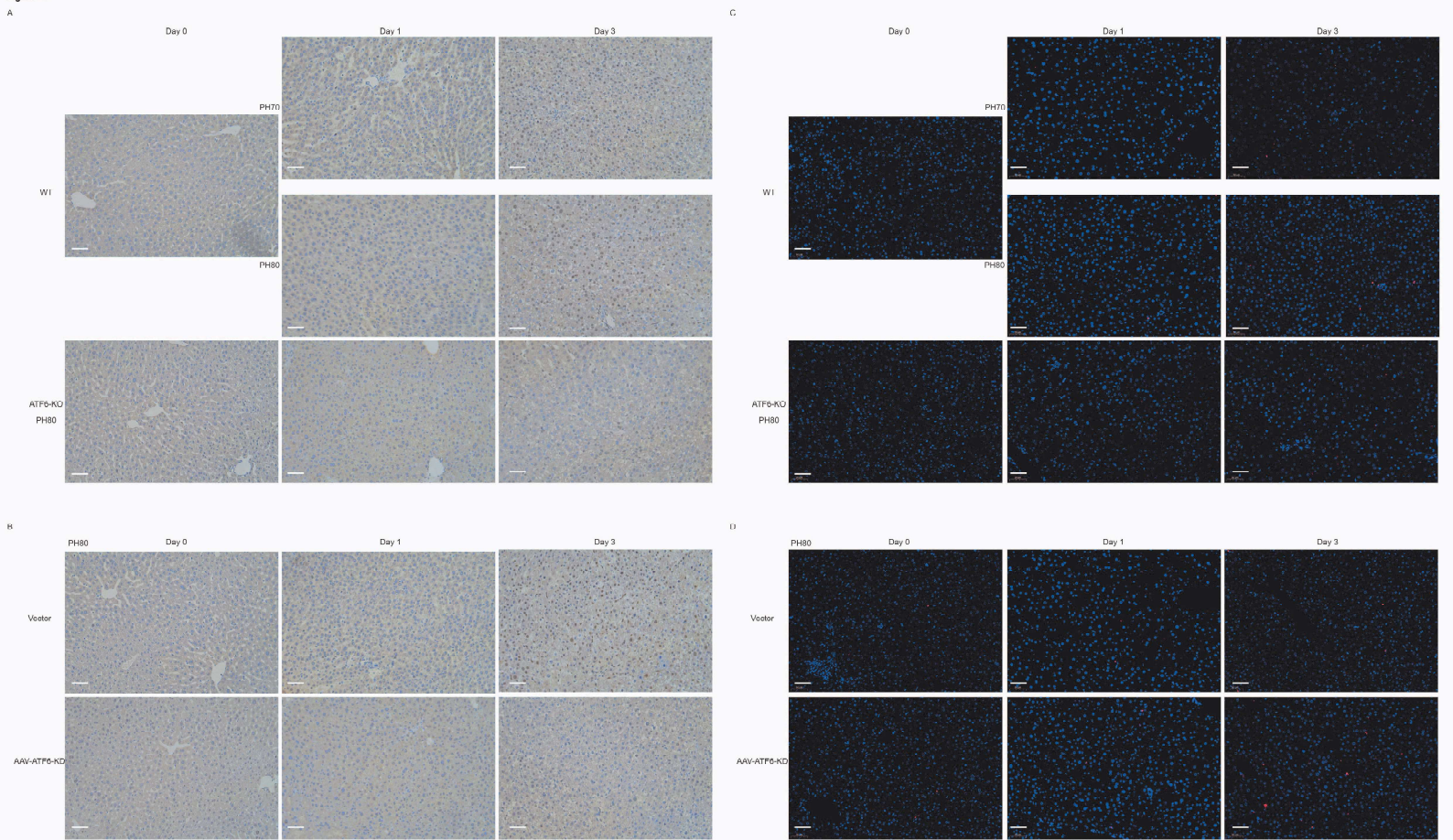

Supplement: Supplementary file 4 — Figure S4: Liver regeneration was assessed by Ki67 IHC staining (A and B), and hepatic apoptosis was detected by TUNEL label staining (C and D) after hepatectomy (original magnification ×400, scale bars: 20 μm). [file FSB2-39-e70933-s004.pdf]
