# Supplementary material for: ATF6 Alleviates Endothelial Inflammation Following Extended Hepatectomy Through Inhibition of TRIM10/NF‐κB Signaling
Source: FASEB J. 2025 Aug 13;39(16):e70933. doi: 10.1096/fj.202402197RRR (PMC12344622; doi:10.1096/fj.202402197RRR)
Supplement: Supplementary file 5 — Table S1: fsb270933‐sup‐0005‐Tables.docx. [file FSB2-39-e70933-s002.docx]

**Supplemental Table**

**Table S1. Clinico-pathologic features of 14 patients with liver cancer**

|  | **Age** (yrs) | **Gender** | **HBV infection** | **Liver cirrhosis** | **Diagnosis*** | **TNM/AJCC Stage** | **Child-pugh stage** | **Major or minor resection** |
| --- | --- | --- | --- | --- | --- | --- | --- | --- |
| **No. 1** | 72 | Male | Positive | Positive | HCC | ⅠB | B | Major resection |
| **No. 2** | 52 | Male | Positive | Positive | HCC | Ⅱ | B | Major resection |
| **No. 3** | 69 | Female | Positive | Positive | HCC | Ⅱ | B | Minor resection |
| **No. 4** | 50 | Male | Positive | Positive | HCC | Ⅱ | A | Major resection |
| **No. 5** | 44 | Male | Positive | Positive | HCC | Ⅱ | A | Major resection |
| **No. 6** | 48 | Male | Positive | Positive | CCC | ⅠB | A | Minor resection |
| **No. 7** | 55 | Male | Positive | Positive | HCC | Ⅱ | B | Minor resection |
| **No. 8** | 62 | Female | Positive | Positive | HCC | Ⅱ | B | Minor resection |
| **No. 9** | 48 | Male | Positive | Positive | HCC | Ⅱ | B | Minor resection |
| **No. 10** | 50 | Male | Positive | Positive | HCC | ⅣA | B | Major resection |
| **No. 11** | 61 | Female | Positive | Positive | CCC | Ⅱ | B | Major resection |
| **No. 12** | 62 | Male | Positive | Positive | HCC | ⅠB | B | Major resection |
| **No. 13** | 50 | Male | Positive | Positive | HCC | Ⅱ | B | Major resection |
| **No. 14** | 55 | Male | Positive | Positive | HCC | Ⅱ | B | Minor resection |

HCC: Hepatocellular Carcinoma; CCC: *Cholangiocellular* Carcinoma.

**Table S2. Primers of quantitative reverse transcription polymerase chain reaction (RT-qPCR)**

| **Gene name** | | **Primer sequence** |
| --- | --- | --- |
| TNF-α | Human | forward 5’-TGGCGTGGAGCTGAGAGATA -3’,  reverse 5’-TGATGGCAGAGAGGAGGTTG -3’ |
|  | Mouse | forward 5’- CAGGCGGTGCCTATGTCTC -3’,  reverse 5’- CGATCACCCCGAAGTTCAGTAG -3’ |
| IL-6 | Human | forward 5’-AGACAGCCACTCACCTCTTCAG -3’,  reverse 5’-TTCTGCCAGTGCCTCTTTGCTG -3’ |
|  | Mouse | forward 5’-TAGTCCTTCCTACCCCAATTTCC -3’,  reverse 5’-TTGGTCCTTAGCCACTCCTTC -3’ |
| β-actin | Human | forward 5’- ACCTTCTACAATGAGCTGCG -3’,  reverse 5’- CCTGGATAGCAACGTACATGG -3 |
|  | Mouse | forward 5’-GGCTGTATTCCCCTCCATCG -3’,  reverse 5’-CCAGTTGGTAACAATGCCATGT -3’ |
| ATF6 | Human | forward 5’- ACCCGTATTCTTCAGGGTGC -3’,  reverse 5’- TCACTCCCTGAGTTCCTGCT -3 |
| TRIM10 | Human | forward 5’- GCAGAGGGAGATGAAGATGTT -3’,  reverse 5’- TGCCATTTGTAGGAGAACTGAG -3 |
| TRIM10  CHIP-PCR | Human | forward 5’- TCAGTGCAGTTACACGCTCTC -3’,  reverse 5’- GGACATGAACTCATCCTTTTTCA -3 |
| Si-ATF6 | Human | Sense 5’- GCACCAUCCCUGAGUCAUUTT-3’,  Antisense 5’- AAUGACUCAGGGAUGGUGCTT-3’ |
| Si-TRIM10 | Human | Sense 5’- CGAGCUCAGUUCUCCUACAAATT-3’,  Antisense 5’- UUUGUAGGAGAACUGAGCUCGTT-3’ |
